# Early changes in gene expression and inflammatory proteins in systemic juvenile idiopathic arthritis patients on canakinumab therapy

**DOI:** 10.1186/s13075-016-1212-x

**Published:** 2017-01-23

**Authors:** Arndt H. Brachat, Alexei A. Grom, Nico Wulffraat, Hermine I. Brunner, Pierre Quartier, Riva Brik, Liza McCann, Huri Ozdogan, Lidia Rutkowska-Sak, Rayfel Schneider, Valeria Gerloni, Liora Harel, Maria Terreri, Kristin Houghton, Rik Joos, Daniel Kingsbury, Jorge M. Lopez-Benitez, Stephan Bek, Martin Schumacher, Marie-Anne Valentin, Hermann Gram, Ken Abrams, Alberto Martini, Daniel J. Lovell, Nanguneri R. Nirmala, Nicolino Ruperto, for the Pediatric Rheumatology International Trials Organization (PRINTO) and the Pediatric Rheumatology Collaborative Study Group (PRCSG)

**Affiliations:** 1 Novartis Institutes for Biomedical Research, Basel, Switzerland; 2 Cincinnati Children’s Hospital Medical Center, PRCSG, Cincinnati, OH USA; 3 Wilhelmina Kinderziekenhuis, Department of Pediatric Immunology and Rheumatology, Utrecht, Netherlands; 4 Université Paris-Descartes, IMAGINE Institute, Hôpital Necker-Enfants Malades. Centre de référence national pour les Arthrites Juveniles, Unité d’Immunologie, Hématologie et Rhumatologie Pediatrique, Paris, France; 5 Rambam Medical Center, Department of Pediatrics B, Haifa, Israel; 6 Alder Hey Children’s NHS Foundation Trust, Liverpool, UK; 7 Cerrahpasa Tip Fakultesi, Ic Hastaliklari ABD, Romatoloji BD, Istanbul, Turkey; 8 Institute of Rheumatology, Paediatric Clinic, Warsaw, Poland; 9 Hospital for Sick Children, Division of Rheumatology, Toronto, ON Canada; 10 Istituto Gaetano Pini, Divisione di Reumatologia, Milano, Italy; 11 Schneider Childrens Medical Center, Pediatric Rheumatology Unit, Petach-Tikvah, Israel; 12 Universidade Federal de São Paulo, Pediatrics, São Paulo, Brazil; 13 British Columbia Children’s Hospital, Vancouver, BC Canada; 14 Universitair Ziekenhuis Gent, Centrum Voor Kinderreumatologie, Gent, Belgium; 15 Randall Children’s Hospital at Legacy Emanuel, Portland, OR USA; 16 Floating Hospital for Children, Rheumatology NEMC 286, Boston, MA USA; 17 Novartis Pharmaceuticals Corporation, East Hanover, NJ USA; 18 University of Genova and Istituto Giannina Gaslini, Pediatria II-PRINTO, Genoa, Italy; 19 Novartis Institutes for Biomedical Research, Cambridge, MA USA; 20 Istituto Giannina Gaslini, Pediatria II-PRINTO, Genoa, Italy

**Keywords:** Biomarkers, Canakinumab, Gene expression, Interleukin-1β, Juvenile idiopathic arthritis, SJIA

## Abstract

**Background:**

Canakinumab is a human anti-interleukin-1β (IL-1β) monoclonal antibody neutralizing IL-1β-mediated pathways. We sought to characterize the molecular response to canakinumab and evaluate potential markers of response using samples from two pivotal trials in systemic juvenile idiopathic arthritis (SJIA).

**Methods:**

Gene expression was measured in patients with febrile SJIA and in matched healthy controls by Affymetrix DNA microarrays. Transcriptional response was assessed by gene expression changes from baseline to day 3 using adapted JIA American College of Rheumatology (aACR) response criteria (50 aACR JIA). Changes in pro-inflammatory cytokines IL-6 and IL-18 were assessed up to day 197.

**Results:**

Microarray analysis identified 984 probe sets differentially expressed (≥2-fold difference; *P* < 0.05) in patients versus controls. Over 50% of patients with ≥50 *a*ACR JIA were recognizable by baseline expression values. Analysis of gene expression profiles from patients achieving ≥50 *a*ACR JIA response at day 15 identified 102 probe sets differentially expressed upon treatment (≥2-fold difference; *P* < 0.05) on day 3 versus baseline, including IL-1β, IL-1 receptors (IL1-R1 and IL1-R2), IL-1 receptor accessory protein (IL1-RAP), and IL-6. The strongest clinical response was observed in patients with higher baseline expression of dysregulated genes and a strong transcriptional response on day 3. IL-6 declined by day 3 (≥8-fold decline; *P* < 0.0001) and remained suppressed. IL-18 declined on day 57 (≥1.5-fold decline, *P* ≤ 0.002).

**Conclusions:**

Treatment with canakinumab in SJIA patients resulted in downregulation of innate immune response genes and reductions in IL-6 and clinical symptoms. Additional research is needed to investigate potential differences in the disease mechanisms in patients with heterogeneous gene transcription profiles.

**Trial registration:**

Clinicaltrials.gov: NCT00886769 (trial 1). Registered on 22 April 2009; NCT00889863 (trial 2). Registered on 21 April 2009.

**Electronic supplementary material:**

The online version of this article (doi:10.1186/s13075-016-1212-x) contains supplementary material, which is available to authorized users.

## Background

Systemic juvenile idiopathic arthritis (SJIA) is a rare, multigenic, autoinflammatory disease of unknown etiology characterized by chronic arthritis; intermittent high-spiking fever, rash, and elevated levels of acute-phase reactants [1–3]. The clinical course is highly variable, ranging from a monocyclic course with a single episode of active disease to the more common persistent clinical course in which the disease remains clinically active. In a minority of patients, the disease follows a polycyclic course, with periods of quiescent disease interrupted by flares of systemic features and mild arthritis [3–5].

SJIA is currently recognized as a distinct subtype of juvenile idiopathic arthritis (JIA), although it is unique among the various forms of JIA in both its epidemiologic and immunopathologic features [1–3]. In contrast to the other JIA phenotypes, which are characterized by abnormalities in adaptive immune response, SJIA is driven by dysregulation of innate immune pathways, with monocytes and neutrophils—rather than lymphocytes—acting as the predominant effector cells [3]. The innate immune signature and clinical features of SJIA, also known as Still’s disease, bear a striking resemblance to adult onset Still’s disease, a rare autoinflammatory disorder characterized by daily spiking temperature, maculopapular rash, and arthritis [6]. Indeed, it has been suggested that these two clinical phenotypes are part of the same disease continuum with different ages of onset [6, 7].

A substantial and growing body of evidence suggests that the pro-inflammatory cytokines interleukins 1, 6, and 18 (IL-1, IL-6, IL-18), and tumor necrosis factor play central roles in the aberrant innate immune response observed in SJIA. In vitro exposure of healthy donor peripheral blood mononuclear cells (PBMCs) to sera from subjects with SJIA induces transcription of genes associated with the IL-1 signaling pathway and markedly increases IL-1β protein secretion [8]. Additionally, treatment of patients with SJIA with agents that block IL-1 signaling pathways resolves clinical symptoms with corresponding improvements in laboratory markers of inflammation [8–14]. Finally, it was recently reported that the proteomics signature associated with disease flares in patients with SJIA is characterized by the differential expression of a small group of proteins that are centrally mediated by IL-1 [15].

Canakinumab is a high-affinity human IL-1β monoclonal antibody that selectively binds to human IL-1β, thereby inactivating IL-1β signaling pathways and neutralizing its downstream effects [16, 17]. Clinical evidence of the efficacy and safety of canakinumab in patients with SJIA was demonstrated in two international phase-3 trials that served as the basis for the approval of canakinumab for the treatment of SJIA by United States and European regulatory authorities [13, 18, 19].

In the present analysis, we sought to characterize the molecular response to treatment with canakinumab and explore potential markers of disease activity and therapeutic response in patients with SJIA using blood-derived biomarker samples from the two phase-3 trials.

## Methods

### Patients

The source population for the biomarker analyses included all patients enrolled in the two phase-3 trials evaluating canakinumab for SJIA (ClinicalTrials.gov, NCT00886769 (trial 1) and NCT00889863 (trial 2)) conducted by the members of the Pediatric Rheumatology International Trials Organization (PRINTO) and the Pediatric Rheumatology Collaborative Study Group (PRCSG) [13]. All patients with available samples who received treatment with either canakinumab or placebo in trial 1 or who were treated with canakinumab in the open-label phase of trial 2 were selected for inclusion in the biomarker study. For selected biomarker analyses, donor samples from age-matched, race-matched, and gender-matched healthy subjects were used as a comparator.

Eligibility criteria and study designs for the original phase-3 trials have been previously described [13]. Briefly, eligible subjects were 2 to 19 years of age with a confirmed diagnosis of SJIA according to published criteria, and who had fever [20]. Concomitant therapy with a prednisone equivalent (up to 1.0 mg/kg/day) and stable doses of nonsteroidal anti-inflammatory drugs and methotrexate (≤20 mg/m^2^ per week) were permitted.

The studies (trials 1 and 2) were conducted according to the ethical principles of the Declaration of Helsinki. The study was approved by the ethics committee of all participating centers according to the local regulations (see SJIA gene expression ethics approval.docx).

### Study design

In trial 1, patients were randomly assigned to receive a single subcutaneous dose of canakinumab (4 mg/kg) or matched placebo. In trial 2, patients were treated with subcutaneous canakinumab (4 mg/kg) every 4 weeks for up to 32 weeks during an initial open-label phase. Patients who achieved a 30 aACR JIA response (defined as improvement of ≥30% in at least three of the six core criteria for JIA, worsening of >30% in no more than one of the criteria, and resolution of fever) and were not receiving glucocorticoids or who had undergone successful tapering and were receiving a stable dose of glucocorticoids, were included in a second withdrawal phase in which they were randomly assigned to continued treatment with canakinumab or placebo.

Blood samples for ribonucleic acid (RNA) isolation were collected at baseline and at day 3 in both trials. For quantification of IL-6 and IL-18, pharmacokinetic serum samples collected at baseline and on days 3, 29, 57, and 197 in trial 2 were used. The parent or guardian of each patient with SJIA and each healthy volunteer provided separate written informed consent (or assent for minors) for the collection and analysis of blood-derived biomarkers.

### Biomarker analyses

#### SJIA gene transcription profile

Global gene transcription was measured in whole blood samples collected from patients with SJIA and age-matched healthy subjects. Microarray analyses were conducted by the Biomarker Development (BMD) group at the Novartis Institutes for Biomedical Research (Basel, Switzerland). Samples were stored at −80 °C and total RNA was subsequently isolated using the PAXgene Blood RNA Kit (Qiagen, Hilden, Germany). Complementary deoxyribonucleic acid (cDNA) was synthesized from total RNA using the Ovation® RNA Amplification System V2 and the Ribo-SPIA® amplification process according to the instructions of the manufacturer (NuGen Technologies Inc., San Carlos, CA, USA). Labeled cDNA was hybridized to GeneChip® U133plus 2.0 arrays as specified by the manufacturer (Affymetrix, Santa Clara, CA, USA). Integrity and purity of RNA and cDNA was confirmed using the Agilent 2100 Bioanalyzer system (Agilent Technologies Inc., Wilmington, DE, USA). Gene expression values were derived using Affymetrix MAS 5.0 software and scaled to a trimmed mean value of 150. Expression values were stored in CEL files and were analyzed using Bioconductor packages or customized R-scripts [21–23].

For group-wise comparison of transcript levels, robustly expressed transcripts were identified by filtering for probe sets with a median expression value of at least 64 in at least one of the groups (typically around 16,000 probe sets). The Wilcoxon test was applied with a threshold *P* value of ≤0.05 to identify genes that were significantly differentially expressed at baseline in patients with SJIA relative to healthy controls. An additional exploratory filtering step for effect size was performed using a 1.5-fold, 2-fold, and 3-fold difference in the median expression value as thresholds. The fold-change cutoffs are arbitrary and were selected before data analysis a priori, and were intended to either describe the more global changes in gene expression or to focus on the most strongly affected genes. Global changes in gene expression were evaluated using the more sensitive threshold of a 1.5-fold difference to facilitate identification of “hits” to signaling pathways (accepting a presumably elevated false positive rate), while the more rigorous threshold of a 3-fold difference was used to identify the most promising biomarker candidates. No correction for multiple comparisons was performed.

Differentially expressed transcripts were displayed using unsupervised two-dimensional hierarchical clustering to group baseline SJIA samples and healthy controls according to gene expression profiles. Expression values were median-centered per gene. Genes with a ≥1.5-fold median increase in baseline SJIA samples relative to normal controls were mapped to protein interaction networks using METACORE™ pathway maps (Thompson Reuters, New York, NY, USA).

#### Early transcriptional response to canakinumab

The early transcriptional response to canakinumab was evaluated by comparing gene expression values in patients with SJIA at day 3 with the values measured at baseline. The magnitude of effect was expressed as the median of the per-patient fold changes in transcription levels at day 3 compared with baseline. Differentially expressed transcripts were identified using the paired sample *t* test (*P* value ≤0.05, ≥1.5-fold differential expression). One-dimensional hierarchical clustering of transcripts was used to display gene expression values ordered according to the aACR JIA response at day 15 to visualize the relationship between baseline gene expression, day 3 transcriptional response, and day 15 clinical response (see Additional file 1: Table S1 for the criteria for each level of response).

The data discussed in this publication have been deposited in the NCBI Gene Expression Omnibus and are accessible through GEO [GEO:GSE80060] (http://www.ncbi.nlm.nih.gov/geo/query/acc.cgi?token=wdyteywqfbuzvgf&acc = GSE80060).

#### Protein biomarkers

A panel of peripheral blood protein markers was selected for analysis based on prior evidence of their association with disease status in patients with SJIA [24–26]. Selected protein markers included IL-6 and IL-18, both of which have been shown to be upregulated in SJIA.

Concentrations of IL-6 and IL-18 were quantified using commercial immunoassay (Quantikine HS Human IL-6 Immunoassay, Catalogue No. SS600B, R&D Systems; Human IL-18 ELISA Kit, Catalogue No. 7620, MBL) validated in human EDTA plasma; three levels of quality controls prepared in human EDTA plasma were used to validate the runs.

#### Sample preparation

Unknown samples (clinical study samples and the human EDTA plasma sample used to prepare quality controls) were stored at −80 °C. On the day of analysis, samples were thawed at room temperature. Once thawed, samples were stored at 2 − 8 °C or on ice if not immediately analyzed. A minimum dilution of 1:5 was applied for the analysis of IL-18.

## Results

Patients with SJIA had a median age of 9 years, 52% were female, and 77% were white, whereas controls had a median age of 9 years, 50% were female, and 91% were white. Patients were characterized by high levels of disease activity at baseline, 99.4% of 177 patients had a juvenile arthritis disease activity score (JADAS)-27 > 8.5, indicating high disease activity [27].

### SJIA gene transcription signature

**Fig. 1:**
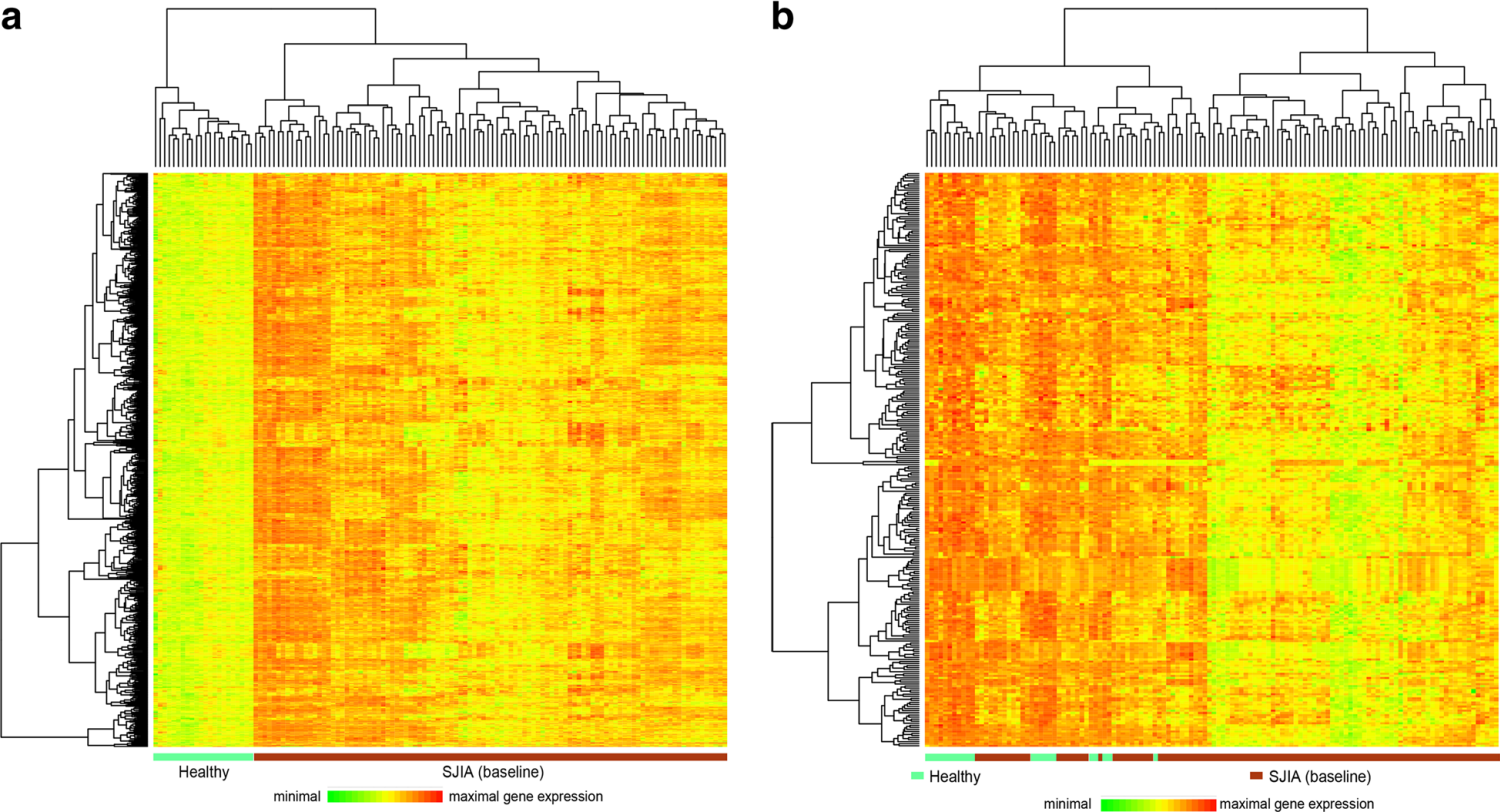
Hierarchical clustering of genes that were upregulated (**a**) and downregulated (**b**) at least twofold in baseline systemic juvenile idiopathic arthritis (*SJIA*) samples compared with healthy control samples. Subjects are shown in *columns* and genes (probe sets) are shown in *rows*. Relative gene expression values are *color*-coded. With upregulated probe sets the hierarchical clustering of subjects perfectly segregated patients with SJIA from healthy controls. Using downregulated genes, the majority of patients with SJIA clustered together; however, a subset of patients with SJIA was indistinguishable from healthy controls

Microarray analysis of whole blood samples collected from patients with SJIA at the baseline study visit identified a total of 984 probe sets with at differential expression at least twofold compared with aged-matched healthy controls. Of these, 704 probe sets were upregulated at least twofold and 280 downregulated at least twofold relative to controls. Unsupervised hierarchical clustering of upregulated genes perfectly discriminated baseline SJIA samples from healthy control samples (Fig. 1a). Clustering of downregulated genes had a more heterogeneous pattern; while the majority of patients with SJIA were grouped in a single cluster that was separated from controls, transcription patterns for downregulated genes in a minority of patients with SJIA were not distinguished from healthy controls (Fig. 1b).

On examination of which genes were more strongly expressed in patients with SJIA, the number of upregulated genes exceeded the number of downregulated genes by nearly 10-fold among the genes with the most marked differences. There were 111 probe sets indicating ≥3-fold upregulation compared with only 12 probe sets indicating ≥3-fold downregulation. Among the strongly upregulated genes were genes that encode neutrophil-expressed proteins like CD177, olfactomedin 4 (OLFM4) or defensin, alpha 4 (DEFA4) and several other regulators of immune function such as tumor necrosis factor, alpha-induced protein 6 (TNFAIP6), IL-1 receptor antagonist (IL1RN), C-type lectin domain family 4 (CLEC4D) or IL-1 receptor-associated kinase 3 (IRAK3); a complete list of strongly differentially expressed genes is available in Additional file 1: Table S2.

**Fig. 2:**
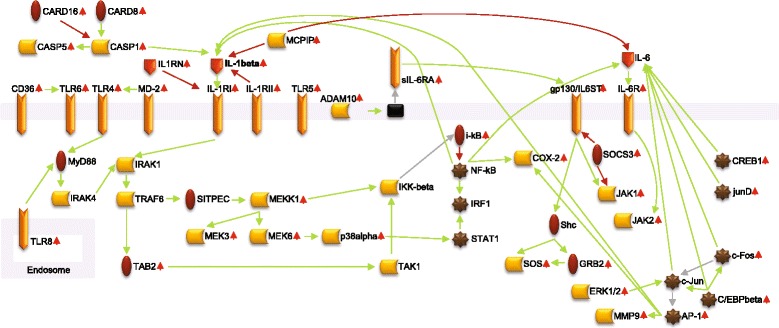
Pathway map for selected proteins encoded by genes that were upregulated in baseline systemic juvenile idiopathic arthritis (*SJIA*) samples. Protein interaction networks for selected proteins that were upregulated in baseline SJIA samples compared with healthy control samples. Proteins were mapped using Metacore™ pathway maps (Thompson Reuters, New York, NY, USA). The network displayed here represents a combination of maps for the signaling pathways for IL-1β, IL-6, and the toll-like receptors TL2 and TL4. *Small red arrows* adjacent to gene/protein names indicate upregulation in SJIA. *ADAM10* disintegrin and metalloproteinase domain-containing protein 10, *AP-1* activator protein 1, *CARD* caspase recruitment domain, *CASP* caspase, *C/EBP CCAAT*/enhancer binding protein, *COX2* cyclooxlygenase-2, *CREB* cyclic AMP responsive element binding protein, *GRB* growth factor receptor-bound protein, *IL-1beta* interleukin-1 beta, *IL-R1* interleukin-1 receptor, *IL1RN* interleukin-1 receptor antagonist, *IL-6* interleukin-6, *IL-6R* interleukin-6 receptor, *sIL-6RA* soluble interleukin-6 receptor antagonist, *IL6ST* interleukin-6 signal transducer, *IRAK* interleukin receptor-associated kinase, *IRF* interferon regulatory factor, *JAK* Janus kinase, *MCPIP* monocyte chemotactic protein-induced protein, *MEK* mitogen activated kinase kinase, *MMP* matrix metalloproteinase, *NFκB* nuclear factor-κB, *SOS* son of sevenless protein, *STAT* signal transducer and activator of transduction, *TAB* TGFβ-activated kinase binding protein, *TAK* TGFβ-activated kinase, *TLR* toll-like receptor

To further examine the functional pathways affected by genes that were differentially expressed in baseline SJIA samples, we mapped SJIA-induced genes (≥1.5-fold differential expression) to protein interaction networks as defined in the METACORE™ database/software. Network maps indicated a strong innate immune response, with widespread activation of inter-related pro-inflammatory signaling pathways. Figure 2 illustrates the interactions between key signaling pathways mediated by selected pro-inflammatory proteins, including IL-1β, IL-6, and the toll-like receptors TLR2 and TLR4.

### Early transcriptional response to canakinumab

**Fig. 3:**
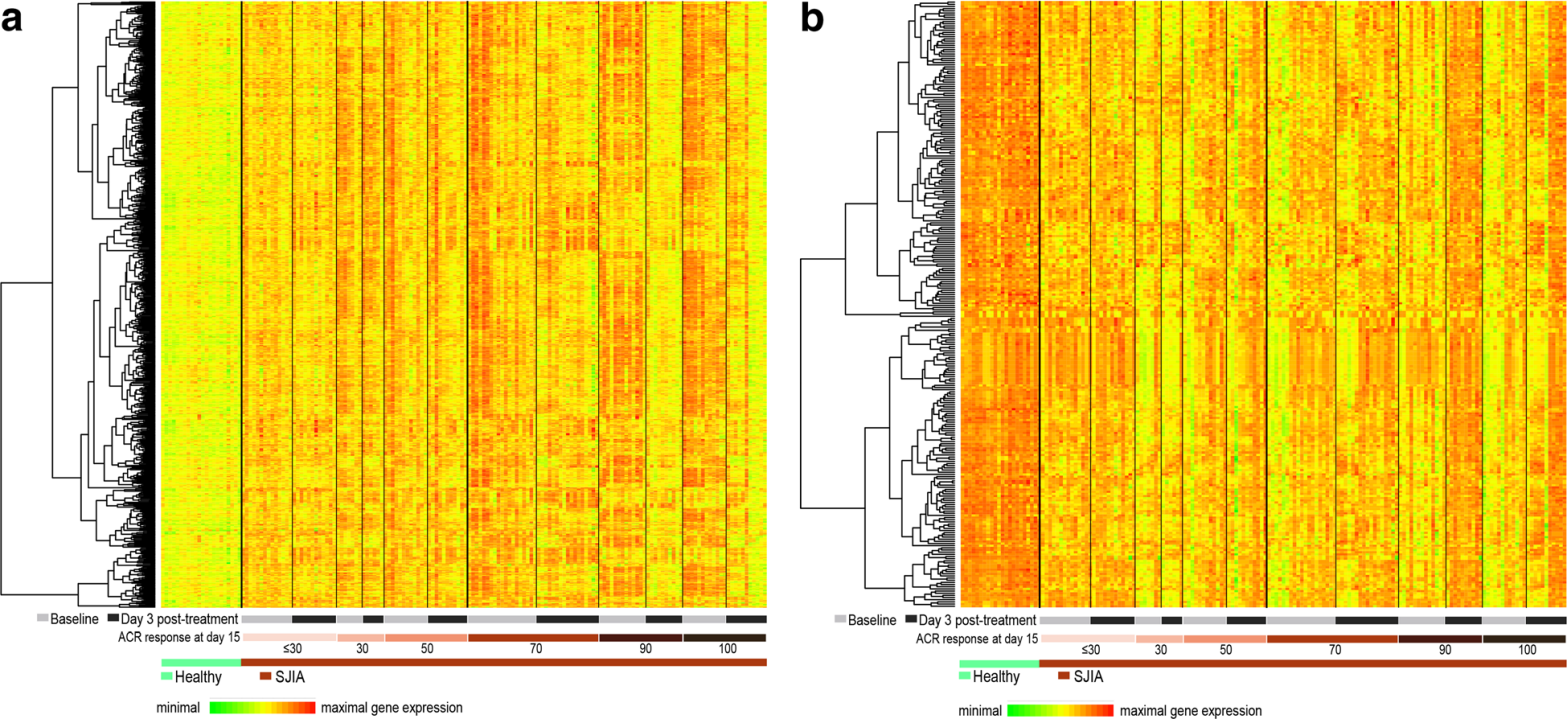
Early transcriptional response to canakinumab treatment of dysregulated genes in systemic juvenile idiopathic arthritis (*SJIA*). Genes (probe sets) are shown in *rows* and subjects are shown in *columns*, ordered according to disease status (SJIA versus healthy), assessment time point (baseline versus day 3), and by response according to American College of Rheumatology (*ACR*) criteria at day 15. Relative gene expression values are *color*-coded. **a** Genes that were upregulated ≥2-fold in baseline SJIA samples compared with healthy control sample. **b** Genes that were downregulated ≥2-fold in baseline SJIA samples compared with healthy control samples

Microarray analysis of samples from patients with SJIA treated with canakinumab and achieving ≥50 aACR JIA response at day 15 identified a total of 1305 probe sets indicating at least 1.5-fold differential expression between baseline and day 3 (no other post-treatment time points were available). These transcripts largely overlapped with those that were dysregulated (in the opposite direction) in baseline SJIA samples as compared with healthy donor samples. The response to canakinumab treatment of SJIA dysregulated genes is shown in Fig. 3, which displays samples ordered by time point (baseline or day 3) and by aACR JIA response score on day 15. These results indicated stronger dysregulation at baseline and also a stronger transcriptional response to canakinumab treatment in a majority of those patients who later (at day 15) reached higher aACR JIA response scores.

Patients who achieved a 100 aACR JIA response at day 15 had high expression of SJIA-induced genes at baseline and a large transcriptional response (downregulation) at day 3, with day-3 values resembling those of healthy controls. In contrast, patients who did not achieve a 30 aACR JIA response on day 15 had weak upregulation of SJIA-induced genes at baseline and essentially unaltered transcription levels on day 3.

Consistent with the observation described, the gene encoding IL-1β—the therapeutic target of canakinumab—also had a robust transcriptional response that was more pronounced in patients who achieved the strongest clinical outcomes. Among patients who did not achieve a 50 aACR, the relative mean gene expression (standard deviation (SD)) at baseline and at day 3 was 894 (501) and 657 (248), respectively. For patients who achieved ≥50 aACR, the relative mean gene expression (SD) at baseline and at day 3 was 1738 (780) and 735 (369), respectively. Transcription levels were also reduced for several IL-1β-related genes, including IL-1RN, IL-1R1, IL-1R2, and IL-1RAP, an essential component of the IL-1 receptor complex that is required for IL-1 signal transduction.

### Protein biomarkers

**Fig. 4:**
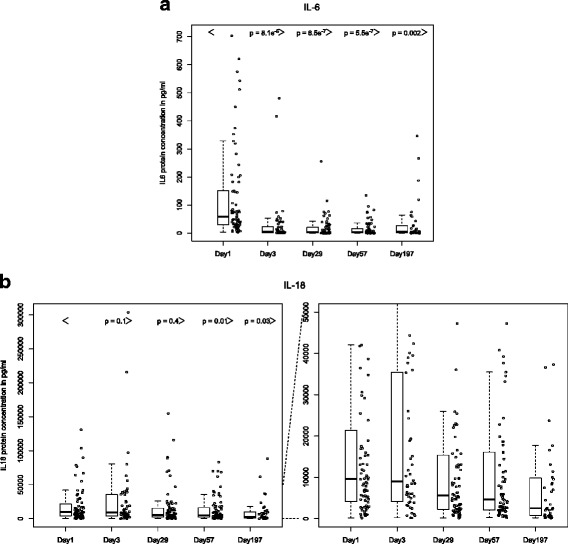
Longitudinal change in serum concentrations of IL-6 (**a**) and IL-18 (**b**) after treatment with canakinumab. Data are shown for trial 2. Similar results were observed for plasma samples in trial 1 (data available until day 29; not shown). The *left panel* (**b**) shows all data; the *right panel* represents a magnification of the lower portion of the *y-axis. IL-6* interleukin-6, *IL-18* interleukin 18

Longitudinal analysis of soluble biomarkers in canakinumab-treated patients showed a marked reduction in serum IL-6 concentration that was evident by the first assessment at day 3 and persisted throughout the duration of observation (Fig. 4a). A more modest reduction was observed for IL-18 serum levels following canakinumab treatment. Additionally, in contrast to IL-6, which declined rapidly following treatment, the reduction in serum concentrations of IL-18 occurred later and was not statistically significant until day 57 (Fig. 4b).

To assess the potential utility of IL-6 and IL-18 as markers of SJIA disease activity status, we compared serum protein levels in patients who achieved a clinical response that was classified as “inactive disease” with those who had clinical evidence of active disease at the corresponding study visit. As per the modified Wallace criteria, inactive disease was defined as absence of active arthritis, fever, and signs or symptoms of SJIA; normal C-reactive protein level; and physician’s global assessment of disease activity ≤10 mm) [28].

Statistically significant differences in serum IL-6 levels were observed at each time point in patients with inactive disease compared with the active disease group. At day 29 the mean IL-6 serum concentration in patients with active disease was 24.3 pg/mL (SD 41.2), whereas in patients with inactive disease the mean concentration was 1.4 pg/mL (SD 1.1) (*P* = 0.0002). Similarly at day 57 the mean IL-6 serum concentration in patients with active disease was 25.2 (SD 31.2), whereas in patients with inactive disease, the mean concentration was 1.5 pg/mL (SD 1.4) (*P* = 8.9e^−6^). At day 197 the mean IL-6 serum concentration in patients with active disease was 45.0 (SD 85.7), whereas in patients with inactive disease, the mean concentration was 1.5 pg/mL (SD 0.5) (*P* = 0.01). Additionally, serum IL-6 levels were normal in all patients classified as having inactive disease; however, normal levels were also observed in patients with active disease. Analysis of serum IL-18 concentrations showed no statistically significant differences between patients with active and inactive disease. At day 29 the mean serum IL-18 concentration in patients with active disease was 14080 pg/mL (SD 26733), whereas in patients with inactive disease, the mean concentration was 21090 pg/mL (SD 30291) (*P* = 0.3). At day 57 the mean IL-18 concentration in the patients with active disease was 10830 pg/mL (SD 16875), whereas in patients with inactive disease the mean concentration was 16790 pg/mL (SD 21661) (*P* = 0.2). At day 197 the mean IL-18 concentration in patients with active disease was 6218 pg/mL (SD 8784), whereas in patients with inactive disease the mean concentration was 7970 pg/mL (SD 12140) (*P* = 0.7).

## Discussion

The current study of blood-derived biomarkers in patients with SJIA treated with canakinumab in a phase-3 clinical trial program yielded several observations with potential implications for patient management and subsequent research. First, analysis of global gene expression in whole blood samples from patients with SJIA and age-matched healthy controls identified robust disease-related alterations of the transcriptome involving key mediators of innate immune response. Consistent with prior research [29–31], differentially expressed genes were typically upregulated relative to healthy controls, supporting the hypothesis that the immune-pathologic process in SJIA is characterized by transcriptional activation of “disease genes” rather than suppression of “health-associated” genes.

Second, we identified a canakinumab response signature in many patients with SJIA that was characterized by the rapid normalization of transcript levels for SJIA overexpressed genes, including those associated with IL-1 and IL-6 signaling pathways. For example, we observed a marked decline in transcription levels of the gene encoding the therapeutic target of canakinumab, IL-1β, suggesting that canakinumab disrupts a positive feedback loop between IL-1β signaling and IL-1β production. The strongest clinical response to canakinumab was observed in patients with higher baseline expression of SJIA-induced genes and a larger transcriptional reduction at day 3; poor clinical response was typically associated with low differential expression of dysregulated genes at baseline and a negligible transcriptional response at day 3.

It is noteworthy that the group of genes comprising the canakinumab response signature in this study closely corresponds with the group of genes that define the transcription signature of adult-onset Still’s disease (AOSD). Indeed, comparison of baseline gene transcription data from patients with AOSD enrolled in a recent trial evaluating canakinumab in healthy adult subjects showed that the mean expression of all of the genes that were downregulated following canakinumab treatment in the current study were upregulated prior to treatment in patients with AOSD relative to healthy controls (*P* < 0.00002) [32, 33]. Similarly, most of the genes that were upregulated following canakinumab treatment in patients with SJIA were downregulated prior to treatment in patients with AOSD compared with healthy controls (*P* < 0.00002). Collectively, these findings support the concept of a Still’s disease continuum that includes both a juvenile-onset and an adult-onset form.

Third, consistent with the early transcriptional effects of canakinumab on genes encoding proteins involved in innate immunity, serum concentrations of the pro-inflammatory cytokines IL-6 and IL-18 declined following treatment with canakinumab. While IL-6 levels declined markedly by day 3 and remained suppressed throughout the duration of treatment, the decline in IL-18 was less robust and occurred much later than the observed declines in IL-6. This might be explained by slow-acting regulatory mechanisms controlling peripheral IL-18 secretion or by residual ongoing pro-inflammatory stimulation of unknown origin. It is important to note that circulating levels of IL-18 have been shown to be markedly elevated in patients with SJIA with macrophage activation syndrome (MAS) [34–36]. While high IL-18 was observed in the three patients who developed MAS during the phase-3 trial of canakinumab, there were quite a few patients with equally high or higher IL-18 who did not develop MAS during the course of the study. Evidence from recent studies suggests a complex interaction between IL-18 and other cytokines in the development of MAS and provides intriguing clues on the potential contribution of free IL-18 levels, the IL-18/IFN-γ ratio, and the IL-18/IL-6 ratio to the prediction of MAS in patients with SJIA [35, 36]. Each of these clues warrants further investigation; however, the limited number of patients with MAS in this cohort precludes meaningful interpretation of the available data.

Fourth, the presence of a number of SJIA-induced genes at baseline, e.g., CD177, OLFM4, and the gene encoding IL-1β, among others, appeared to lead to a stronger transcriptional response to canakinumab treatment and higher ACR responses at day 15. However, in a significant proportion of canakinumab responders, these transcriptional effects were not observed, and therefore, baseline gene expression would not correctly predict good canakinumab responders. The predictive performance of baseline gene expression, with respect to other previously identified markers of response to IL-1 inhibition, will be the subject of future investigation.

The findings of the present study should be interpreted in the context of certain limitations. First, while the observed changes in gene expression were presumably influenced by changes in the cellular composition of the samples, the precise contribution of changes in blood cell populations to the observed transcriptional response cannot be reliably ascertained. Additionally, the degree to which the findings might have been influenced by factors such as disease duration and severity, prior treatment with other biologic therapies, and concomitant exposure to corticosteroids during the study is unknown. Second, baseline gene transcription was evaluated using the single pre-treatment blood sample that was available for each patient. Given the dynamic nature of the disease with frequent spikes in fever, it is possible that the differences between patients with SJIA and healthy controls were attenuated or obscured and that the contrast in gene transcription profiles might be enhanced by integrating data from multiple pre-treatment measurements. This might also reduce the apparent heterogeneity observed in subgroups of patients who achieved a comparable clinical response to canakinumab. Finally, patients without fever and with a more prominent polyarticular disease course were excluded from enrollment in the original phase-3 clinical trials from which blood samples for the current study were obtained. Accordingly, the effect of canakinumab on gene expression in patients with SJIA with a polyarticular disease course is unknown.

## Conclusions

Our findings demonstrate that canakinumab treatment in patients with SJIA results in rapid reductions in the expression of genes related to inflammation, with corresponding reductions in circulating levels of pro-inflammatory cytokines and clinical symptoms, suggesting that canakinumab can, at least in part, reverse molecular disease patterns.

## Additional file

Additional file 1:
**Table S1** Definition of adapted ACR JIA response. **Table S2** Genes with ≥3-fold differential expression between patients with SJIA and healthy controls, prior to canakinumab treatment. **Table S3** List of independent ethics committees or institutional review boards (trial 1). **Table S4** List of independent ethics committees or institutional review boards (trial 2). (DOCX 53 kb)

## Abbreviations

aACR: adapted American college of rheumatology
ACR: American College of Rheumatology
BMD: biomarker development
cDNA: complimentary deoxyribonucleic acid
CLEC4D: C-type lectin domain family 4
DEFA4: defensin, alpha 4
IL: interleukin
IL1-R: interleukin-1 receptor
IL1-RAP: interleukin-1 receptor accessory protein
IL1RN: interleukin-1 receptor antagonist
IRAK3: interleukin-1 receptor-associated kinase 3
JADAS: juvenile arthritis disease activity score
JIA: juvenile idiopathic arthritis
OLFM4: olfactomedin 4
PBMCs: peripheral blood mononuclear cells
PRCSG: Pediatric Rheumatology Collaborative Study Group
PRINTO: Pediatric Rheumatology International Trials Organization
RNA: ribonucleic acid
SJIA: systemic juvenile idiopathic arthritis
TNFAIP6: tumor necrosis factor, alpha-induced protein 6
