# Supplementary material for: Early changes in gene expression and inflammatory proteins in systemic juvenile idiopathic arthritis patients on canakinumab therapy
Source: Arthritis Res Ther. 2017 Jan 23;19:13. doi: 10.1186/s13075-016-1212-x (PMC5260050; doi:10.1186/s13075-016-1212-x)
Supplement: Additional file 1: — Table S1 Definition of adapted ACR JIA response. Table S2 Genes with ≥3-fold differential expression between patients with SJIA and healthy controls, prior to canakinumab treatment. Table S3 List of independent ethics committees or institutional review boards (trial 1). Table S4 List of independent ethics committees or institutional review boards (trial 2). (DOCX 53 kb) [file 13075_2016_1212_MOESM1_ESM.docx]

**Early Changes in Gene Expression and Inflammatory Proteins in Systemic Juvenile Idiopathic Arthritis Patients on Canakinumab Therapy**

Arndt H. Brachat, Alexei A. Grom, Nico Wulffraat, Hermine I Brunner, Pierre Quartier, Riva Brik, Liza McCann, Huri Ozdogan, Lidia Rutkowska-Sak, Rayfel Schneider,^9^Valeria Gerloni, Liora Harel, Claudio Len, Kristin Houghton, Rik Joos, Daniel Kingsbury, Jorge M. Lopez-Benitez, Stephan Bek, Martin Schumacher, Marie-Anne Valentin, Hermann Gram, Ken Abrams, Alberto Martini, Daniel J. Lovell, Nanguneri R. Nirmala, Nicolino Ruperto for the Pediatric Rheumatology International Trials Organization (PRINTO) and the Pediatric Rheumatology Collaborative Study Group (PRCSG).

**Data Supplement**

**Table S1** **Definition of adapted ACR pediatric response**

The adapted ACR pediatric response is a composite measure that includes the following seven response variables:

1. Physician’s Global Assessment of disease activity on a 0-100 mm VAS

2. Global Assessment of Patient’s overall wellbeing (0-100 mm VAS in the CHAQ©)

3. Functional ability: CHAQ^©^

4. Number of joints with active arthritis

5. Number of joints with limitation of motion

6. Laboratory measure of inflammation: CRP (mg/L)

7. Absence of intermittent fever due to SJIA during the preceding week

Response is defined according to various levels of percentage improvement from baseline (30%, 50%, 70%, 90%) in at least three of the first six response variables (core criteria) and no intermittent fever (temperature >38°C) in the preceding week (variable 7), with no more than one of the first six variables worsening more than 30%.

For example, an **adapted** **ACR 30** **response** is defined as an improvement of at least **30%** in at least three of the first six variables, with no intermittent fever in the preceding week and no more than one of the first variables worsening more than **30%**.

| **Adapted ACR Response** | **Criteria** |
| --- | --- |
| ACR 30 | - ≥30% improvement in at least 3 of 6 core criteria - >30% worsening in no more than 1 core criterion - No intermittent fever during the last 7 days |
| ACR 50 | - ≥50% improvement in at least 3 of 6 core criteria - >30% worsening in no more than 1 core criterion - No intermittent fever during the last 7 days |
| ACR 70 | - ≥70% improvement in at least 3 of 6 core criteria - >30% worsening in no more than 1 core criterion - No intermittent fever during the last 7 days |
| ACR 90 | - ≥90% improvement in at least 3 of 6 core criteria - >30% worsening in no more than 1 core criterion - No intermittent fever during the last 7 days |
| ACR 100 | - ≥100% improvement in at least 3 of 6 core criteria - >30% worsening in no more than 1 core criterion - No intermittent fever during the last 7 days |

**Table S2 Genes showing a ≥3-fold differential expression between SJIA patients and healthy controls prior to canakinumab treatment**

| **Gene symbol** | **Affymetrix probe set** | **Fold change (SJIA vs. healthy)** | ***P* value*** |
| --- | --- | --- | --- |
| CD177 | 219669_at | 28.6 | 3.87E-09 |
| OLFM4 | 212768_s_at | 10.1 | 1.17E-07 |
| SNCA | 236081_at | 9.9 | 9.42E-12 |
| MMP8 | 231688_at | 8.8 | 1.76E-09 |
| DEFA4 | 207269_at | 6.9 | 4.40E-06 |
| CA1 | 205950_s_at | 6.9 | 1.57E-09 |
| ARHGEF12 | 233621_s_at | 6.3 | 8.68E-09 |
| CRISP3 | 207802_at | 6.3 | 4.29E-10 |
| ARG1 | 206177_s_at | 6.2 | 1.05E-09 |
| MMP9 | 203936_s_at | 5.9 | 3.39E-11 |
| CEACAM8 | 206676_at | 5.6 | 1.83E-06 |
| INSC | 237056_at | 5.0 | 3.08E-10 |
| MS4A4A | 219607_s_at | 5.0 | 1.13E-10 |
| - | 239210_at | 5.0 | 6.72E-10 |
| - | 238403_at | 4.9 | 5.70E-09 |
| - | 224051_at | 4.8 | 6.29E-12 |
| MARCKS | 201668_x_at | 4.8 | 6.88E-12 |
| TNFAIP6 | 206025_s_at | 4.7 | 8.24E-10 |
| CLEC4D | 1552773_at | 4.6 | 9.12E-11 |
| SLC8A1 | 1561615_s_at | 4.6 | 1.53E-12 |
| IL1RN | 216243_s_at | 4.6 | 1.95E-10 |
| ITGB3 | 204627_s_at | 4.6 | 2.21E-10 |
| CLEC4D | 1552772_at | 4.6 | 2.61E-11 |
| IRAK3 | 1568830_at | 4.6 | 3.56E-13 |
| MS4A4A | 224357_s_at | 4.4 | 3.39E-11 |
| PROS1 | 207808_s_at | 4.3 | 8.37E-11 |
| ANKRD22 | 238439_at | 4.1 | 2.22E-08 |
| OSBP2 | 223432_at | 4.1 | 2.39E-08 |
| ITGB3 | 204628_s_at | 4.1 | 2.31E-12 |
| GPR84 | 223767_at | 4.1 | 2.83E-09 |
| CYP1B1 | 202435_s_at | 4.0 | 1.29E-11 |
| ARG1 | 231662_at | 4.0 | 5.22E-11 |
| PLXNC1 | 206470_at | 4.0 | 4.96E-13 |
| F5 | 204713_s_at | 4.0 | 6.88E-12 |
| PF4V1 | 207815_at | 4.0 | 2.98E-08 |
| - | 206302_s_at | 3.9 | 2.03E-10 |
| RNF182 | 230720_at | 3.9 | 0.002245 |
| MYL9 | 201058_s_at | 3.9 | 9.01E-09 |
| ALPL | 1557924_s_at | 3.9 | 4.66E-10 |
| ANXA3 | 209369_at | 3.8 | 4.29E-08 |
| CPEB4 | 224829_at | 3.8 | 7.23E-13 |
| - | 242094_at | 3.8 | 2.61E-11 |
| - | 241916_at | 3.8 | 5.71E-10 |
| PGLYRP1 | 207384_at | 3.7 | 1.52E-10 |
| HP | 206697_s_at | 3.7 | 1.51E-06 |
| C12orf39 | 229778_at | 3.7 | 1.09E-09 |
| CALD1 | 212077_at | 3.7 | 3.18E-09 |
| REPS2 | 242571_at | 3.7 | 8.74E-11 |
| CYP1B1 | 202436_s_at | 3.7 | 3.65E-12 |
| SLC14A1 | 205856_at | 3.7 | 1.57E-09 |
| C19orf59 | 235568_at | 3.6 | 5.33E-08 |
| SNCA | 204467_s_at | 3.6 | 1.69E-09 |
| - | 206655_s_at | 3.5 | 2.96E-10 |
| VNN1 | 205844_at | 3.5 | 1.28E-09 |
| LIN7A | 241652_x_at | 3.5 | 3.64E-10 |
| TNFAIP6 | 206026_s_at | 3.5 | 2.62E-09 |
|  |  |  |  |
| SOX6 | 228214_at | 3.5 | 3.71E-08 |
| CACNA1E | 236013_at | 3.5 | 1.01E-07 |
| TDRD9 | 228285_at | 3.5 | 1.65E-10 |
| KCNH7 | 224099_at | 3.5 | 2.39E-11 |
| LTF | 202018_s_at | 3.5 | 4.68E-05 |
| VNN1 | 1558549_s_at | 3.5 | 2.83E-09 |
| ITGA2B | 206494_s_at | 3.4 | 5.48E-10 |
| THBS1 | 201108_s_at | 3.4 | 5.95E-10 |
| - | 214807_at | 3.4 | 1.65E-10 |
| FECH | 203115_at | 3.4 | 1.60E-07 |
| IRAK3 | 220034_at | 3.4 | 1.67E-12 |
| TET2 | 235461_at | 3.4 | 6.90E-13 |
| - | 238405_at | 3.4 | 6.38E-08 |
| FKBP5 | 204560_at | 3.3 | 1.92E-12 |
| BASP1 | 228589_at | 3.3 | 5.75E-12 |
| YOD1 | 227309_at | 3.3 | 1.61E-11 |
| FAM210B | 224693_at | 3.3 | 2.21E-10 |
| ITGA2B | 206493_at | 3.3 | 7.29E-10 |
| DSC2 | 226817_at | 3.3 | 4.70E-09 |
| SULT1B1 | 207601_at | 3.3 | 5.00E-11 |
| EGF | 206254_at | 3.3 | 9.42E-12 |
| TMEM158 | 213338_at | 3.3 | 1.47E-11 |
| PLBD1 | 222639_s_at | 3.2 | 6.90E-13 |
| WDFY3 | 212598_at | 3.2 | 2.50E-10 |
| LOC731424 | 1559777_at | 3.2 | 4.14E-08 |
| CPEB4 | 224828_at | 3.2 | 2.96E-10 |
| MAP2K6 | 205699_at | 3.2 | 1.52E-10 |
| C9orf84 | 1553920_at | 3.2 | 7.36E-11 |
| IL1RN | 212659_s_at | 3.2 | 2.24E-09 |
| LIN7A | 240027_at | 3.2 | 1.95E-10 |
| - | 226162_at | 3.2 | 8.73E-13 |
| IL1RN | 216245_at | 3.2 | 8.24E-10 |
| F5 | 204714_s_at | 3.2 | 1.61E-11 |
| VCAN | 215646_s_at | 3.2 | 4.39E-11 |
| MCTP1 | 1554730_at | 3.2 | 5.48E-10 |
| - | 239464_at | 3.2 | 1.28E-10 |
| CYP1B1 | 202437_s_at | 3.2 | 1.95E-10 |
| HIPK3 | 207764_s_at | 3.2 | 1.28E-09 |
| PFKFB2 | 209992_at | 3.1 | 4.96E-08 |
| LCN2 | 212531_at | 3.1 | 5.97E-06 |
| YBX3 | 228634_s_at | 3.1 | 7.06E-11 |
| ARHGAP6 | 206167_s_at | 3.1 | 1.23E-10 |
| CD274 | 227458_at | 3.1 | 0.000434 |
| - | 238488_at | 3.1 | 5.94E-08 |
| BPI | 205557_at | 3.1 | 3.45E-08 |
| SLC8A1 | 241752_at | 3.1 | 2.84E-11 |
| GADD45A | 203725_at | 3.1 | 1.46E-12 |
| FKBP5 | 224856_at | 3.1 | 3.24E-11 |
| - | 208470_s_at | 3.1 | 8.58E-06 |
| XK | 206698_at | 3.1 | 3.71E-08 |
| PBX1 | 212151_at | 3.1 | 2.07E-09 |
| - | 239205_s_at | 3.0 | 4.51E-13 |
| CPD | 201942_s_at | 3.0 | 1.84E-12 |
| ITGA2B | 216956_s_at | 3.0 | 2.40E-10 |
| SOCS3 | 206359_at | 3.0 | 4.59E-11 |
| - | 222347_at | -3.1 | 1.74E-05 |
| HLA-DQB1 | 212999_x_at | -3.3 | 0.000152 |
| LRRN3 | 209841_s_at | -3.3 | 1.61E-11 |
| FCRL5 | 224404_s_at | -3.4 | 3.90E-07 |
| LRRN3 | 209840_s_at | -3.6 | 4.00E-12 |
| HLA-DQB1 | 209480_at | -3.8 | 0.002446 |
| ALOX15 | 207328_at | -3.8 | 2.78E-07 |
| - | 236203_at | -3.9 | 0.018511 |
| - | 213831_at | -3.9 | 0.012313 |
| TCL1A | 39318_at | -4.0 | 1.99E-09 |
| TCL1A | 209995_s_at | -4.0 | 1.99E-09 |
| - | 1556402_at | -4.4 | 5.95E-10 |

*Wilcoxon test

**Table S3 List of Independent Ethics Committees or Institutional Review Boards (Trial 1)**

| **Center No.** | **Ethics Committee or Institutional Review Board** |
| --- | --- |
| 0003 | Comité de Ética del Hospital Pedro Elizalde |
| 0011 | Comitê de ética em pesquisa do Hospital Universitário Pedro Ernesto |
| 0015 | Comitê de ética em pesquisa em seres humanos do Hospital de Clinicas UFPR |
| 0020 | UZ Leuven-Ethics Committee |
| 0021 | UZ-Gent Ethics Committee |
| 0022 | UCS Saint Luc-Comite d'Ethque |
| 0023 | HUDERF |
| 0040 | CPP-Ile de France II |
| 0041 | CPP-Ile de France II |
| 0051 | Ethik-Kommission |
| 0052 | Ethik-Kommission |
| 0058 | Ethik-Kommission |
| 0060 | Geschäftsstelle der Ethik-Kommission des Landes Berlin |
| 0066 | Ethik-Kommission |
| 0070 | Medical Research Council |
| 0080 | Kaplan Ethics Committee |
| 0081 | The Chaim Sheba Ethics Committee |
| 0082 | Meir Sapir EC |
| 0083 | Rambam EC |
| 0096 | Comitato Etico per le Attività Biomediche dell'Università degli Studi Federico II di Napoli |
| 0115 | Medisch Ethisch Toetsingscommissie |
| 0120 | Komisja Bioetyczna przy Instytucie Reumatologii |
| 0130 | Ceic Hospital Sant Joan de Deu |
| 0132 | CEIC Hospital La Fe |
| 0135 | Regionala etikprövningsnämnden i Stockholm |
| 0140 | Commission d'Ethiquo de la recherche clinique |
| 0145* | Dokuz Eylul University Faculty of Medicine Ethics Committee (December 2008 – 26 January 2010)  Central Independent Ethics Review Board (11 March 2010 – 1 September 2010)  Istanbul University Medical Facility (Since November 2010) |
| 0146* | Dokuz Eylul University Faculty of Medicine Ethics Committee (December 2008 – 26 January 2010)  Central Independent Ethics Review Board (11 March 2010 – 1 September 2010)  Istanbul University Medical Facility (Since November 2010) |
| 0147* | Dokuz Eylul University Faculty of Medicine Ethics Committee (December 2008 – 26 January 2010)  Central Independent Ethics Review Board (11 March 2010 – 1 September 2010)  Istanbul University Medical Facility (Since November 2010) |
| 0148* | Dokuz Eylul University Faculty of Medicine Ethics Committee (December 2008 – 26 January 2010)  Central Independent Ethics Review Board (11 March 2010 – 1 September 2010)  Istanbul University Medical Facility (Since November 2010) |
| 0155 | Comité Institucional de Ética en Investigación de la Universidad San Martin de Porres - Clínica CADA MUJER |
| 0200 | Central London REC 2 |
| 0204 | Central London REC 2 |
| 0205 | Central London REC 2 |
| 0220 | General Hospital of Thessaloniki Ippokratio |
| 0501 | Institutional Review Board |
| 0503 | Western Institutional Review Board |
| 0506 | Legacy Clinical Research and Technology Center |
| 0508 | University of Louisville Med Center One |
| 0510 | University of Arkansas for Medical Sciences |

*During 2010 the Turkish clinical research regulations went through several changes and therefore more than one ethics committee oversaw the CACZ885G2305 study. During periods where there was no formal ethics committee, the Turkish Ministry of Health allowed ongoing Ministry of Health approved studies to continue for the safety and interest of the volunteer patients.

**Table S4 List of Independent Ethics Committees or Institutional Review Boards (Trial 2)**

| **Center No.** | **Ethics Committee or Institutional Review Board** |
| --- | --- |
| 0003 | Comite Independiente de Etica para Ensayos de Farmacologia Clinica |
| 0011 | COMITÊ DE ÉTICA EM PESQUISA DO HOSPITAL UNIVERSITÁRIO PEDRO HERNESTO - UERJ |
| 0012 | COMITÊ DE ÉTICA EM PESQUISA DA UNIVERSIDADE FEDERAL DE SÃO PAULO – HOSPITAL SÃO PAULO |
| 0015 | COMITÊ DE ÉTICA EM PESQUISA EM SERES HUMANOS DO HOSPITAL DE CLÍNICAS / UFPR |
| 0016 | COMITÊ DE ÉTICA EM PESQUISA DO INSTITUTO DE PUERICULTURA E PEDIATRIA MARTAGÃO GESTEIRA – CEP/IPPMG |
| 0020 | UZ Leuven – Ethische Commissie |
| 0021 | UZ Gent – Ethisch Commissie |
| 0022 | UCL Saint-Luc – Comité d'Ethique |
| 0023 | HUDERF - Comité d'Ethique |
| 0030 | Comité d’Éthique de la Recherché – Centre Hospitalier Universitaire Ste-Justine |
| 0031 | UBC (Univertisy of British Columbia) C&W (Children’s & Women’s Health Centre of British Columbia) Research Ethics Board |
| 0034 | The Hospital for Sick Children Research Ethics Board |
| 0040 | Comité de Protection des Personnes "Ile de France II" |
| 0041 | Comité de Protection des Personnes "Ile de France II" |
| 0042 | Comité de Protection des Personnes "Ile de France II" |
| 0043 | Comité de Protection des Personnes "Ile de France II" |
| 0051 | Ethik-Kommission |
| 0052 | Ethik-Kommission |
| 0057 | Ärztekammer Hamburg |
| 0058 | Ethikkommission der |
| 0059 | Ethik-Kommission des Landes Berlin |
| 0060 | Ethik-Kommission des Landes Berlin |
| 0066 | Ethik-Kommission der |
| 0070 | Medical Research Council |
| 0080 | Kaplan Helsinki comittee |
| 0081 | Sheba Helsinki comittee |
| 0082 | Meir Helsinki comittee |
| 0083 | Rambam Helsinki comittee |
| 0084 | Rabin Helsinki comittee |
| 0090 | IRCCS Istituto Giannina Gaslini |
| 0093 | A.O.Istituto Ortopedico Gaetano Pini-Università degli studi |
| 0094 | A.O.U. Anna Meyer |
| 0096 | A.Osped.-Universit. Policlinico Federico II Univ.degli Studi |
| 0099 | Fond.IRCCS Ca' Granda Osp.Maggiore Policlinico |
| 0110 | REK sør-øst |
| 0115 | METC UMC |
| 0120 | Komisja Bioetyczna przy Instytucie Reumatologii |
| 0130 | Comite Etico de Investigacion Clinica |
| 0131 | Comite Etico de Investigacion Clinica |
| 0132 | Comite Etico de Investigacion Clinica |
| 0133 | Comite Etico de Investigacion Clinica |
| 0135 | Regionala etikprovningsnamnden i Stockholm |
| 0140 | Commission cantonale d'ethique de la recherche sur l'etra humain |
| 0145 | Dokuz Eylul University Faculty of Medicine Clincal Researchs Local Ethical Committee |
| 0146 | Dokuz Eylul University Faculty of Medicine Clincal Researchs Local Ethical Committee |
| 0147 | Dokuz Eylul University Faculty of Medicine Clincal Researchs Local Ethical Committee |
| 0148 | Dokuz Eylul University Faculty of Medicine Clincal Researchs Local Ethical Committee |
| 0155 | Comité Institucional de Ética en Investigación de la Universidad San Martin de Porres - Clínica CADA MUJER |
| 0200 | NHS National Research Ethics Service |
| 0202 | NHS National Research Ethics Service |
| 0204 | NHS National Research Ethics Service |
| 0205 | NHS National Research Ethics Service |
| 0206 | NHS National Research Ethics Service |
| 0207 | NHS National Research Ethics Service |
| 0215 | Ethikkommision der Medizinischen Universität Wien und des Allgemeinen Krankenhauses der Stadt Wien AKH |
| 0220 | National Ethics Committee |
| 0501 | Office of Research Compliance & Regulatory Affairs |
| 0503 | Western Institutional Review Board |
| 0506 | Legacy Health System and Institutional Review Board |
| 0508 | Human Subjects Protection Program |
| 0510 | Institutional Review Board, Office of Research and Sponsored Programs |
| 0512 | Institutional Review Board |
| 0513 | Committee on Clinical Investigations |
